# Supplementary material for: Museum Genomics Reveals Temporal Genetic Stasis and Global Genetic Diversity in Arabidopsis thaliana
Source: Mol Ecol. 2025 Aug 13;34(20):e70081. doi: 10.1111/mec.70081 (PMC12530293; doi:10.1111/mec.70081)
Supplement: Supplementary file 1 — Figures S1–S5: mec70081‐sup‐0001‐FiguresS1‐S5.docx. [file MEC-34-e70081-s001.docx]

**Supplemental Material**

*DNA extraction, library preparation and sequencing: Newly generated data*

*Herbarium samples –* One hundred and thirty newly obtained samples from herbaria were transported into a dedicated ancient DNA laboratory at Pennsylvania State University, USA. To avoid introducing contamination, the plastic bags containing the samples were cleaned with a 2% bleach solution. The surface of each sample was gently cleaned of potential soil and dust particles with a thin brush. The brush was cleaned in between samples by submerging the tip in a 2% bleach solution and then thoroughly rinsing it with milli-Q water (Millipore Corporation). DNA extraction was done using one medium-size leaf of *Arabidopsis thaliana*. Prior to extraction, tissue was homogenized using a TissueLyser (Qiagen). Each sample followed two cycles of homogenization, the first cycle was done for 30 seconds and the second was 15 seconds to ensure all tissue had been pulverized. Then, the DNA was extracted using the PTB protocol recommended for ancient samples with minor modifications (Wales & Kistler, 2019). Modifications were made to the length of the digestion (24 hours), to the final amount of Buffer AW1 (5x) and the amount of the elution buffer (25 µl). After extraction, DNA concentration was determined in a Qubit fluorometer using the Qubit dsDNA high-sensitivity (HS) Assay Kit (ThermoFisher Scientific). In addition, 5 µl of DNA from each sample were run in a 1% agarose gel to visually inspect the degree of DNA fragmentation.

After DNA extraction, we proceeded to library preparation. DNA obtained from herbarium samples is expected to be highly fragmented. Thus, no shearing step is done before library preparation. However, since our study includes both samples from recent years (~ 10 years ago) and samples collected one or two centuries ago, we performed library preparation based on the particular fragment-size of each sample. Samples that showed DNA above the 500 bp ladder band were subjected to a short shearing step (30 seconds) using a M220 Focused Ultrasonicator (Covaris). Samples lacking DNA above the 500 bp ladder band were processed for library preparation as recommended for ancient samples (no shearing step). Herbarium-preserved samples are known to show fragmentation and damage rapidly after preservation (Staats et al., 2011). Thus, to capture short fragments and maximize complexity despite low input concentration we followed a library preparation method that relies on blunt-end ligation of custom adapters (Meyer & Kircher, 2010). This method is known to be more efficient and less-biased for chemically damaged and fragmented DNA than the AT-overhang adapter ligation commonly used in commercially available library preparation kits (Seguin-Orlando et al., 2013). One modification was implemented for the non-sheared samples; cleaning steps during the library preparation process were done using first the QIAquickNucleotide Removal Kit (Qiagen) and then the MinElute PCR Purification Kit (Qiagen) instead of AMPure XP/SPRIselect magnetic beads (Beckman Coulter). Final DNA content in the libraries was quantified using a Qubit fluorometer with the Qubit dsDNA high-sensitivity (HS) Assay Kit (ThermoFisher Scientific). Since samples had individual identifying barcodes, a pooled equimolar aliquot was prepared for sequencing. Each sequencing run contained approximately 40 randomly selected samples, including extraction and library preparation blanks to ensure that no contamination had happened during any step. The 42 samples used for the pilot study were sequenced PE at 100bp in a HiSeq 2500 platform. The remaining samples were sequenced at 75bp PE in a NextSeq 550 Illumina sequencer.

*Recent field collections -* A total of 95 fresh samples were included in this study (61 field samples from Norway (McKay et al., 2025), 30 from East Africa and 4 from the INRA stock center). Thirteen fresh leaf samples were collected from the field in East Africa while the remaining 17 were collected as seeds. Leaf material was immediately desiccated in silica gel within sealed plastic bags. The samples were transported to the laboratory and stored at 4ºC until DNA extraction. Seed samples from East Africa, together with seeds from the 4 INRA stock center lines, were cultivated in growth chambers, and leaf tissue was dried in silica gel. Prior to DNA extraction all samples were flash-frozen in liquid Nitrogen and then homogenized as done with the herbarium samples. Next, DNA was extracted using the DNeasy Plant Mini Kit (Qiagen) following the manufacturer's recommendations. The extracted DNA concentrations were determined in a Qubit fluorometer using the Qubit dsDNA high-sensitivity (HS) Assay Kit (ThermoFisher Scientific). DNA from the field-collected leaf samples were sent to BGI (China) for library preparation and sequencing. These samples were sequenced with paired end (PE) 150bp reads on a HiSeq X Ten Illumina machine. The remaining samples were processed by Genomics and Bioinformatics Service at Texas A&M AgriLife, where library preparation was conducted and sequencing was done on a NovaSeq 6000 Illumina platform with PE 150bp reads. Finally, the Norwegian field samples were collected as seeds and cultivated in a Colorado State University greenhouse. A single plant was sampled from each family once plants reached the vegetative state, and leaf tissue was from that plant was used for subsequent DNA extraction using the Qiagen DNeasy Plant Mini Kit (Valencia CA, USA). Extracted DNA was then quantified using a Qubit Fluorometer (ThermoFisher Scientific). Whole genome sequencing (WGS) libraries of the extracted DNA were prepared at the University of Colorado Boulder sequencing core. These WGS libraries were then paired-end (2 x 150 bp) whole genome sequenced at the University of Colorado Anschutz Medical Campus using an Illumina HiSeq.

*Data preprocessing and* de novo *SNP calling*

*Herbarium samples preprocessing* - Herbarium samples were mostly newly generated data (130). To these we added five samples from the African genomes (Durvasula et al., 2017) and 33 German genomes (Lang et al., 2024) also collected from herbarium vouchers. Raw data for the African genomes was obtained from the European Nucleotide archive (ENA) of the European Molecular Biology Lab - European Bioinformatics Institute (EMBL - EBI) and German genomes from (Lang et al., 2024). Raw read sequence data were assessed with FastQC (http://www.bioinformatics.babraham.ac.uk/projects) to confirm that they met our quality standards. For all herbarium samples cytosine deamination profiles characteristic of ancient DNA were verified using mapDamage 2.0 (Jónsson et al., 2013) (Figure S1). Samples processed using the traditional aDNA library preparation and samples sheared before library preparation were checked with MapDamage 2.0 separately to ensure that all meet the ancient DNA criteria. Reads were trimmed using Leehom with the -ancientdna flag (Renaud et al., 2014). Merged and unmerged reads were then concatenated to maximize genome coverage. Trimmed reads were mapped to the *Arabidopsis thaliana* nuclear genome (TAIR v. 10) with the BWA v.0.7.17 -*aln* function since this option has a higher mapping rate for short fragments compared with alternatives (Li, 2013; Li & Durbin, 2009). After the reads from each sample were mapped to the nuclear genome, SAM_TOOLS_ v.1.5 was used to transform the mapped SAM files to BAM, sort the BAM files, remove duplicates, and filter the reads (minimum mapping quality of 30 and 30 base pair minimum length) (Li et al., 2009). Finally, SAM_TOOLS_ *flagstat* was used to count the number of alignments for every sample mapped to the *A. thaliana* nuclear genome (Li et al., 2009).

*Fresh samples and publicly available data preprocessing* - Raw data from 199 samples (Alonso-Blanco et al., 2016) were downloaded from the Sequence Read Archive (SRA) of the National Center for Biotechnology Information (NCBI). Additionally, the 65 African samples (all Moroccan except one from Tanzania) were obtained from the European Nucleotide archive (ENA) of the European Molecular Biology Lab - European Bioinformatics Institute (EMBL - EBI). For all the publicly available samples and the ones generated from fresh tissue, raw read sequence data were assessed with FastQC (http://www.bioinformatics.babraham.ac.uk/projects) to confirm that they met our quality standards. Raw paired-end reads were trimmed and filtered using Trimmomatic-0.38 to remove adapter sequences, short reads, and low-quality reads from raw sequence data (Bolger et al., 2014). To remove adapter sequences, we used seed matches with a maximum of three mismatches and an initial length of 10. These seeds were extended and clipped when paired-end reads had a score of 30 or lower. Additionally, leading and trailing bases of low quality (Phred quality score <20) or N were removed. Likewise, reads where the quality of a 4-base-wide sliding window dropped below 15 at the 3’ end were removed. Finally, any remaining reads that were shorter than 75 bases long were eliminated. Trimmed reads were then mapped to the *Arabidopsis thaliana* nuclear genome (TAIR v. 10) with BWA v.0.7.17 -*mem* flag (Li, 2013; Li & Durbin, 2009). After the reads from each sample were mapped to the nuclear genome, SAM_TOOLS_ v.1.5 was used to convert the mapped SAM files to BAM, sort the BAM files, remove duplicates, and filter the reads (minimum mapping quality of 30 and 30 base pair minimum length) (Li et al., 2009). Finally, SAM_TOOLS_ -*flagstat* was used to count the number of alignments for every sample mapper to the *A. thaliana* nuclear genome (Li et al., 2009).

de novo *SNP calling -* SNP discovery was done using a pseudohaplotype approach (Kistler et al., 2018). A combination of SAM_TOOLS_ *mpileup* and VarScan v2.3.9 *mpileup2snp* was used to discover all candidate variant positions in all samples with minimum mapping quality of 20, a minimum read depth of 2 and at least 2 supporting reads to call a variant (Koboldt et al., 2009; Li et al., 2009). To exclude paralogs and repetitive regions, variable sites in the top 10% of coverage were filtered out and the remaining sites were summarized in a bed file. The bed file was used to create individual pseudohaplotype files for each sample of variable sites with a minimum coverage of 2 and a maximum of 70 (these coverage thresholds were selected based on the specific coverage data from all samples included in this study). After filtering sites by coverage, individual files were combined into plink tped and tfam files and culled to biallelic sites. Finally, PLINK v1.90 was used create the final vcf file containing the selected variable sites (Purcell et al., 2007).

*Simulating purifying and background selection*

We used the SLiM simulation software to simulate a Wright-Fisher model with semi-realistic genome structure (genes with introns and exons, and intergenic space) and a combination of neutral and deleterious mutations, with deleterious mutations only occurring in genic sequences (Haller & Messer, 2019). We simulated a stepping-stone model of 10 populations with a migration rate of 0.005 and used a selfing rate of 95%. We ran models for 100k generations, and tested simulations with different combinations of genome size, deleterious mutation distribution of fitness effects, and recombination rates. We then tested each SNP of at least 5% MAF in a sample of 200 individuals across the last 100 generation in regression models with time (as in the Arabidopsis data). We then tested for enrichment of the temporal allele frequency change statistics for mutations in different genomic contexts (genic vs intergenic).

Figure S1: Historical sample authentication. Merged (on the left) and unmerged reads (on the right) were run separately. Black indicates samples that were not sheared (aDNA) and red indicates samples that were shared (sheared) before library preparation due to differences among samples in the existing degree of fragmentation. A) Fraction of C-to-T converted base-pairs along sequencing reads and the B) correlation of C-to-T conversion at the first base of a read respective to sample’s age. These patterns show the characteristic damage done to DNA fragments accumulated over time.


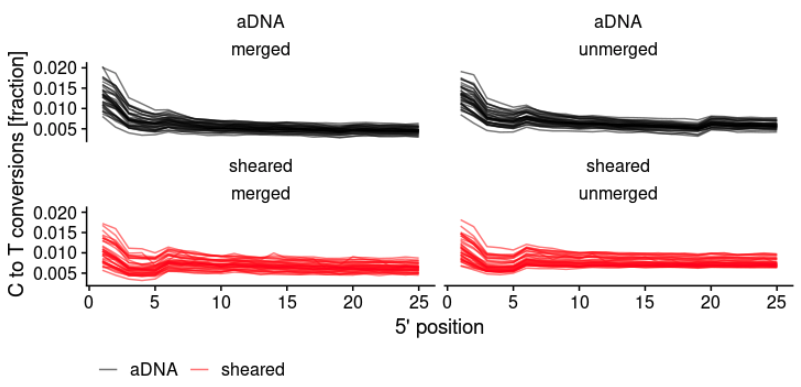


b)

a)


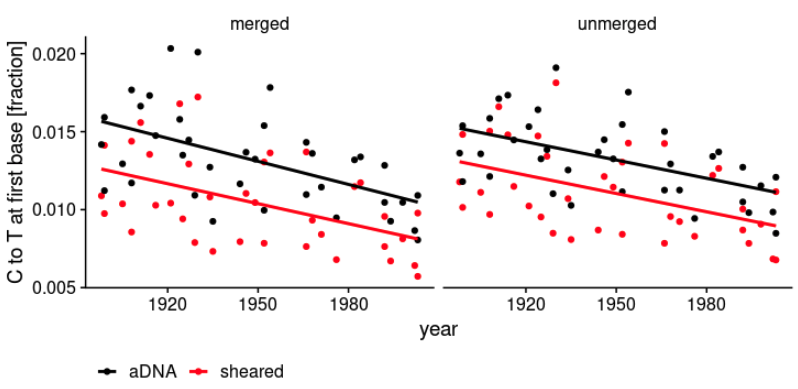


Figure S2. ADMIXTURE results for K=2-15, with samples grouped along the x-axis by geographic region as in Figure 1. Colors indicate assignment to ancestral clusters.


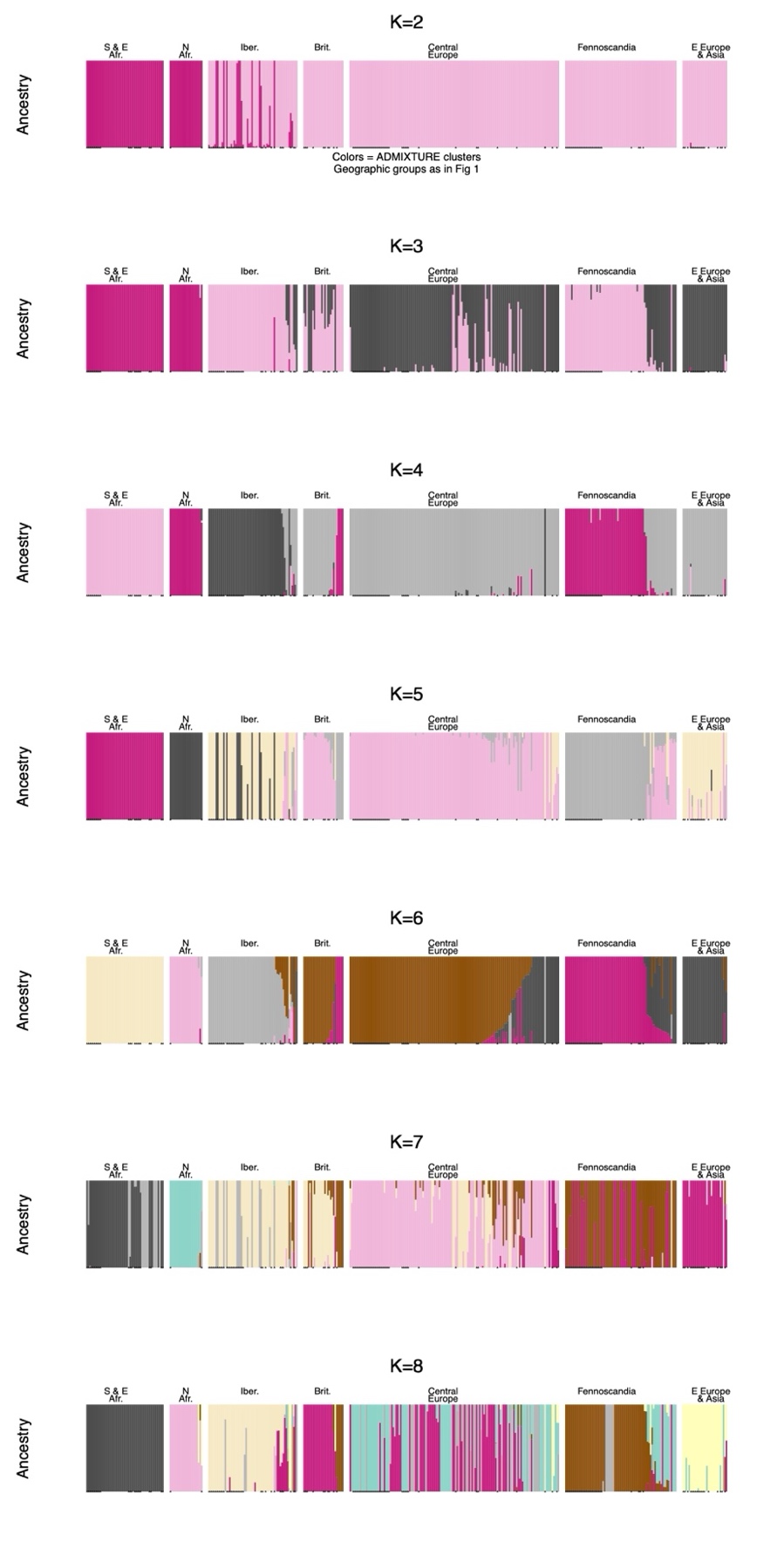


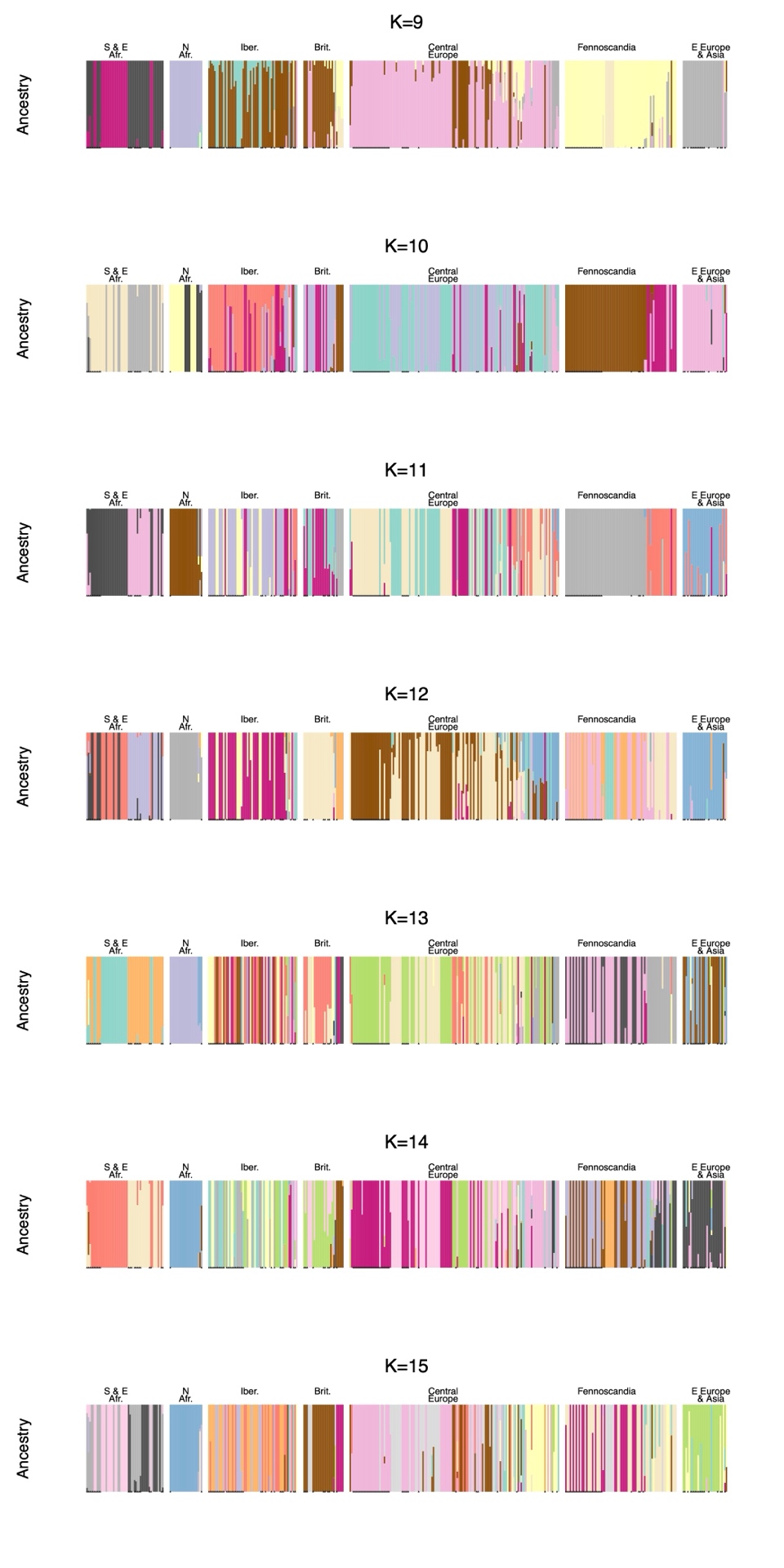


Figure S3. Results of ADMIXTURE clustering with different K values **across Eurasia**. This clustering was primarily for visualization purposes and determining turnover in genotypes within regions in Figure 4. For this reason, we used K=6 in analyses, though we also conducted a version of analysis with K=3. This K resolved different regional genotypic clusters but came before a sharp increase in cross validation error at K=7.


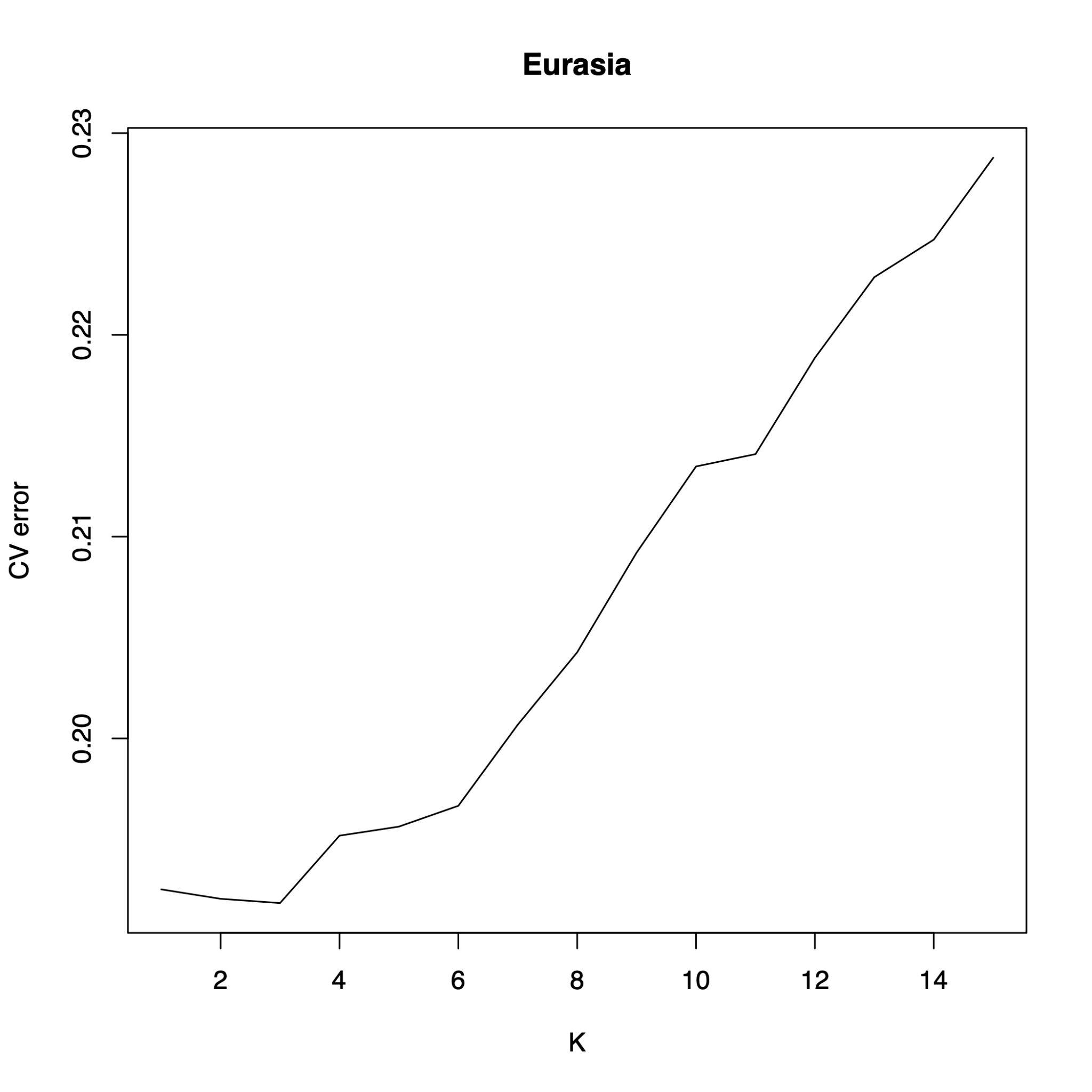


Figure S4. Comparing the observed difference in temporal allele shifts in conserved (‘cons’) versus non-conserved (‘nc’) genes of different lengths (AA = amino acid) for Eurasian samples. For each gene we calculated the 0.1 quantile p-value for SNPs in the gene, and then we compared the median of these values between conserved and non-conserved genes. When the observed is *less* than zero and the null, it indicates conserved genes show greater temporal allele frequency shifts (as for genes less than or equal to 200 AA in length using the Wilcoxon and logistic tests that do not correct for population structure). When the observed is *greater* than zero and the null, it indicates non-conserved genes show greater temporal allele frequency shifts (as for genes greater than 600 AA in length). The overall trends are similar between tests except for small genes, where the greater turnover in conserved small genes is only seen in tests that do not correct for population structure (Wilcoxon and logistic) while the mixed model turnover is not different for small, conserved genes compared to non-conserved. This discrepancy for small genes suggests that the high turnover for conserved small genes follows the genome-wide pattern for turnover, a turnover possibly due to drift being stronger due to their background selection. However, this hypothesis does not seem consistent with what is observed for longer conserved genes (less turnover than expected).

Figure S5. Results from simulations of purifying and background selection show no clear effect on the enrichment of genic SNPs for temporal allele frequency change. Purifying selection acts on a subset of coding SNPs and a subset of intronic SNPs, with neutrality for other SNPs. The y-axes show the lower tail of a permutation based null-model test, where the test statistic is the genic SNPs’ 0.01 p-value quantile minus the intergenic SNPs’ 0.01 quantile (1^st^ and 3^rd^ columns) or 0.05 quantile (2^nd^ and 4^th^ columns). Both linear mixed models testing year correlations with SNPs while accounting for kinship (1^st^ and 2^nd^ columns), or simple logistic year versus SNP allele (3^rd^ and 4^th^ columns) models are shown. Dots show individual simulation results, lines show means.
